# Preparation of Nafion/Polycation Layer-by-Layer Films for Adsorption and Release of Insulin

**DOI:** 10.3390/polym10080812

**Published:** 2018-07-24

**Authors:** Kentaro Yoshida, Katsuhiko Sato, Tetsuya Ono, Takenori Dairaku, Yoshitomo Kashiwagi

**Affiliations:** 1 School of Pharmaceutical Sciences, Ohu University, 31-1 Misumido, Tomita-machi, Koriyama, Fukushima 963-8611, Japan; t-ono@pha.ohu-u.ac.jp (T.O.); t-dairaku@pha.ohu-u.ac.jp (T.D.); y-kashiwagi@pha.ohu-u.ac.jp (Y.K.); 2 Graduate School of Pharmaceutical Sciences, Tohoku University, Aramaki, Aoba-ku, Sendai 980-8578, Japan; satok@m.tohoku.ac.jp

**Keywords:** layer-by-layer film, controlled release, insulin, Nafion, drug delivery

## Abstract

Thin films were prepared using layer-by-layer (LbL) deposition of Nafion (NAF) and polycations such as poly(allylamine hydrochloride) (PAH), poly(ethyleneimine) (PEI), and poly(diallydimethylammonium chloride) (PDDA). Insulin was then adsorbed on the NAF-polycation LbL films by immersion in an insulin solution. The NAF-polycation LbL films were characterized using a quartz crystal microbalance and an atomic force microscope. The release of insulin from the LbL films was characterized using UV-visible adsorption spectroscopy and fluorescence emission spectroscopy. The greatest amount of insulin was adsorbed on the NAF-PAH LbL film. The amount of insulin adsorbed on the (NAF/PAH)_5_NAF LbL films by immersion in a 1 mg mL^−1^ insulin solution at pH 7.4 was 61.8 µg cm^−2^. The amount of insulin released from the LbL films was higher when immersed in insulin solutions at pH 2.0 and pH 9.0 than at pH 7.4. Therefore, NAF-polycations could be employed as insulin delivery LbL films under mild conditions and as an insulin release control system according to pH change.

## 1. Introduction

Layer-by-layer (LbL) films can be prepared by alternate and repeated deposition of a polyelectrolyte on a solid surface through electrostatic interactions [1,2]. The driving force for the construction of LbL films is not only electrostatic interactions but also interactions such as hydrogen bonding [3,4], avidin-biotin binding [5], and sugar-lectin binding [6,7]. The materials employed for this purpose include synthetic polymers [8,9,10], proteins [11], polysaccharides [12,13], and DNA [14]. Such layered thin films have found applications in separation and purification [15,16], sensors [17,18,19], microcapsules [20,21], and drug delivery systems (DDSs) [22,23]. Attempts have been made to develop an insulin-release LbL based DDS that responds to pH [24,25,26] and sugar levels [27,28,29,30].

We have recently prepared insulin-containing LbL films for the pH-triggered release of insulin [26]. This LbL film has an advantage in that it can be prepared with ease and without the use of organic solvents. The LbL film can be prepared not only as flat films but also as microparticles; therefore there is a wide choice of substrates on which to coat the LbL film [25,31]. The adsorption of insulin onto the LbL film is due to the electrostatic interaction between the net electrical charges of insulin and the counter-polymer in the LbL film. However, satisfactory results were not obtained with respect to the amount of adsorbed insulin. Therefore, for the adsorption of insulin onto an LbL film we attempted using Nafion (NAF) as a cation exchange polymer.

**Figure 1:**
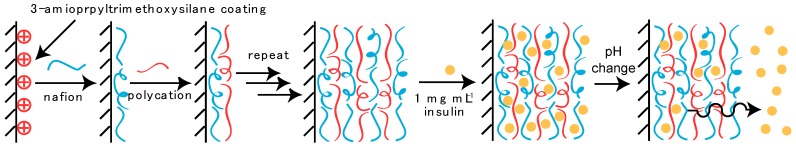
Schematic illustration for the preparation of a Nafion (NAF)-based layer-by-layer (LbL) film and insulin release from the LbL film in media of differing pH.

NAF, a perfluorosulfonic acid membrane that consists of a hydrophobic Teflon backbone and side chains that contain hydrophilic sulfonic groups, is a copolymer [32]. NAF film possesses chemical stability and high proton conductivity, and has thus attracted much attention as an electrolyte material for proton exchange membrane fuel cells [33,34]. NAF has a high selectivity for cation exchange; however, there have been few studies on the use of NAF as a material for controlled drug release. NAF could be used as a breakthrough drug reservoir material by facilitating the adsorbion of the drug onto a thin film. The release of a drug adsorbed onto an LbL thin film with NAF is expected to occur through a change in the net charge of the drug corresponding to a change in the pH. Here we report on the preparation of an LbL film with NAF and a polycation, the adsorption of insulin by immersion of the LbL film in insulin solution, and the subsequent pH-dependent insulin release, as shown in Figure 1.

## 2. Experimental Section

### 2.1. Materials

Nafion^®^ (NAF; DE1021 CS type) and insulin (human, recombinant) were purchased from Wako Pure Chemical Industry (Osaka, Japan). Poly(allylamine hydrochloride) (PAH; *M_w_*:70,000), poly(ethyleneimine) (PEI), poly(diallydimethylammonium chloride) (PDDA; *M_w_*:100,000–200,000), poly(styrene sulfate) (PSS; *M_w_*:500,000), and poly(vinylsufonate) (PVS; *M_w_*:240,000) were acquired from Nittobo Co. (Tokyo, Japan), Tokyo Kasei Industry Co. (Tokyo, Japan), Sigma-Aldrich Co. (St. Louis, MO, USA), Scientific Polymer Product, Inc. (New York, NY, USA), and Nacalai Tesque Co. (Kyoto, Japan), respectively. Fluorescein isothiocyanate-labeled insulin (FITC-insulin) was synthesized by the coupling reactions of insulin and fluorescein isothiocyanate (FITC) [29]. All other reagents were of the highest grade and used without further purification.

### 2.2. Preparation of LbL Films

A quartz crystal microbalance (QCM; 420E QCM BAS Co., Tokyo, Japan) was employed for gravimetric analysis of the LbL films consisting of NAF and PAH, PEI, and PDDA. An 8 MHz AT-cut quartz resonator coated with a gold (Au) layer (geometric surface area, 0.20 cm^2^) was used as a probe, in which the deposition of 1 ng of substrate induces a ca. −0.75 Hz change in the resonance frequency. The Au surface layer of the quartz resonator was cleaned using piranha solution (a mixture of H_2_O_2_ and H_2_SO_4_, 1:3 *v*/*v*) and thoroughly rinsed in pure water before use (CAUTION: Piranha solution should be handled with extreme care). The cleaned quartz resonator was immersed in an aqueous solution of cystamine dihydrochloride (5 mM) for 12 h to make a positively charged surface on the Au surface layer. After rinsing the surface of the cystamine-modified quartz resonator, it was exposed to a solution of 0.5 mg mL^−1^ NAF in 10 mM Tris buffer containing 150 mM NaCl (pH 7.4) for 15 min to deposit the first NAF layer, and then rinsed with the working buffer for 5 min. The NAF-deposited quartz resonator was then exposed to 0.5 mg mL^−1^ polycation solution in the Tris buffer (pH 7.4) for 15 min and then rinsed with the Tris buffer solution for 5 min. The deposition of NAF and polycation was repeated to prepare the LbL films. For the purpose of recording frequency data, the film-deposited probe was rinsed with milli-Q water for 1 min and then dried in air until the frequency showed a steady-state value. All data for the dry film were obtained in air.

### 2.3. Adsorption of Insulin to LbL Film

A quartz slide (50 × 9 × 1 mm) was first treated in a 1% solution of 3-aminopropyltrimethoxysilane in methanol overnight to make the surface positively charged. The slide was then washed with milli-Q water and heated at 60 °C. The surface of positively charged slides was coated with LbL film by immersing the slide alternately in 0.5 mg mL^−1^ polyanion (PVS, PSS, and NAF) and 0.5 mg mL^−1^ polycation (PAH, PEI, and PDDA) in 10 mM Tris buffer containing 150 mM NaCl (pH 7.4) for 15 min. The LbL film prepared on a quartz slide was then exposed to a 0.1 mg mL^−1^ or a 1 mg mL^−1^ insulin solution with 10 mM acetate buffer (pH 2) or 10 mM Tris buffer (pH 7.4) for 12 h, followed by rinsing with the working buffer solution for 5 min. All buffer solutions contained 150 mM NaCl. UV-visible absorption spectra of insulin adsorbed on the LbL films in the working buffer were recorded on a UV-visible adsorption spectrometer (V-560, Jasco Co., Tokyo, Japan). The LbL films immersed in insulin solution were exposed to sodium hydroxide solution for 5 h, and the absorbance of the solution was recorded to determine the concentration of insulin loading onto the LbL films.

### 2.4. pH-Dependent Release of FITC-Insulin

The pH-dependent release of FITC-insulin from LbL films was studied using fluorescence emission spectroscopy (P-6500, Jasco Co., Tokyo, Japan). The LbL film prepared on a quartz slide was exposed to 0.1 mg mL^−1^ FITC-insulin solution (pH 7.4) for 12 h and then rinsed with the working buffer for 5 min. FITC-insulin adsorbed LbL films were exposed to 10 mM acetate buffer at pH 2.0–4.0 or 10 mM Tris buffer at pH 7.4–9.0 (all buffer solutions contained 150 mM NaCl), and the fluorescence intensity of the exposed solution was recorded to determine the concentration of FITC-insulin released from the LbL films. 

### 2.5. Statistics

The data were expressed as means ± standard error of mean (SEM). Comparisons were made using a one-way analysis of variance (ANOVA) and a Dunnett’s test. A *p*-value of less than 0.05 was considered significant. 

## 3. Results and Discussion

**Figure 2:**
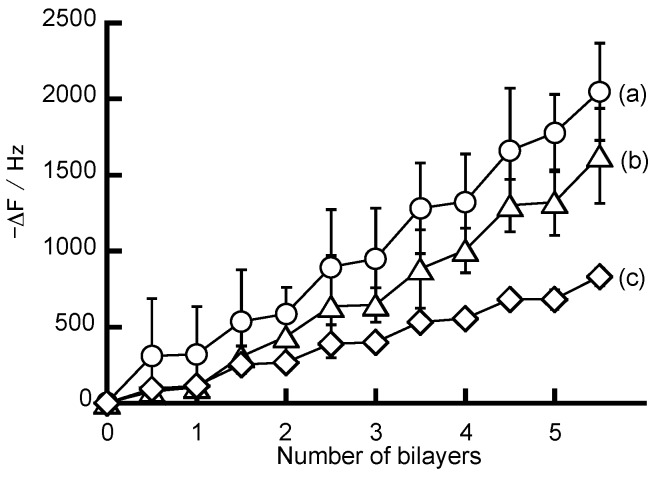
QCM frequency change for the deposition of (**a**) Nafion poly(allylamine hydrochloride) (NAF-PAH); (**b**) Nafion poly(ethyleneimine) (NAF-PEI); and (**c**) Nafion poly(diallyldimethylammonium chloride (NAF-PDDA) LbL films.

Figure 2 shows the QCM results for the preparation of NAF-PAH, NAF-PEI, and NAF-PDDA films. The change in the resonance frequency (ΔF) was recorded after drying in air to exclude the possible effect of bound water on the ΔF value. The value of −ΔF was increased when the quartz resonator was exposed to NAF and PAH, PEI, or PDDA, which indicated that NAF-PAH, NAF-PEI, and NAF-PDDA films were successfully formed on the surface of the quartz resonator. It is considered that negatively charged NAF and positively charged PAH, PEI, and PDDA are deposited by electrostatic attraction, which builds up the LbL film on the quartz resonator surface. The adsorption of 1 ng of the substance on the quartz resonator induced a −0.75 Hz change in ΔF. From the QCM data, the amount of deposited LbL film was calculated to be 14.6 µg cm^−2^ for the (NAF/PAH)_5_NAF film, 11.6 µg cm^−2^ for the (NAF/PEI)_5_NAF film, and 5.9 µg cm^−2^ for the (NAF/PDDA)_5_NAF film. The difference in the amount of LbL film adsorption on the quartz resonator surface was estimated according to the structure of the polycation. PAH is a linear molecule composed of a larger number of primary amine groups (-NH_2_) than PEI, and has a particular affinity for NAF. In contrast, the amount of deposition was decreased when PDDA was used. PDDA is a rigid molecule with extended chains that lead to intramolecular repulsion, causing it to have a lower affinity for NAF than both PAH and PEI.

**Figure 3:**
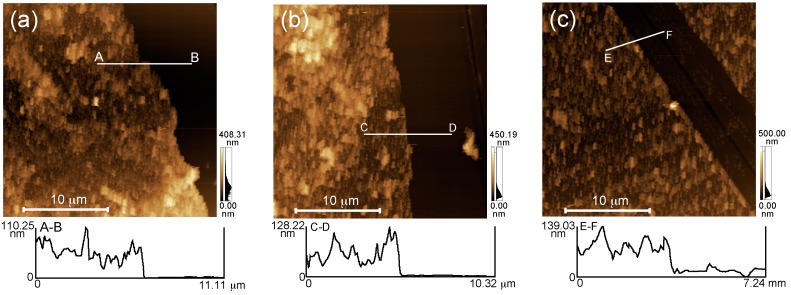
Atomic force microscopy (AFM) images of the (**a**) (NAF/PAH)_5_NAF; (**b**) (NAF/PEI)_5_NAF; and (**c**) (NAF/PDDA)_5_NAF films.

Figure 3 shows atomic force microscopy (AFM; SPM-9600, Shimadzu, Kyoto, Japan) images of dried (NAF/polycation)_5_NAF films. The glass slides used to prepare each of the LbL films were rinsed with milli-Q water, and dried for 24 h in a desiccator. The thicknesses of the LbL films were determined by scratching the film-coated glass slide using a cutter and conducting AFM scans over the scratch. AFM images were acquired in contact mode at room temperature in air. The surface roughness and thicknesses of the LbL films were estimated to be 72.94 ± 26.88 nm for the (NAF/PAH)_5_NAF film, 55.29 ± 16.67 nm for the (NAF/PEI)_5_NAF film, and 43.74 ± 20.12 nm for the (NAF/PDDA)_5_NAF film. The height difference in the thicknesses of the LbL films was dependent on the amount of deposition, obtained from the QCM measurements.

**Figure 4:**
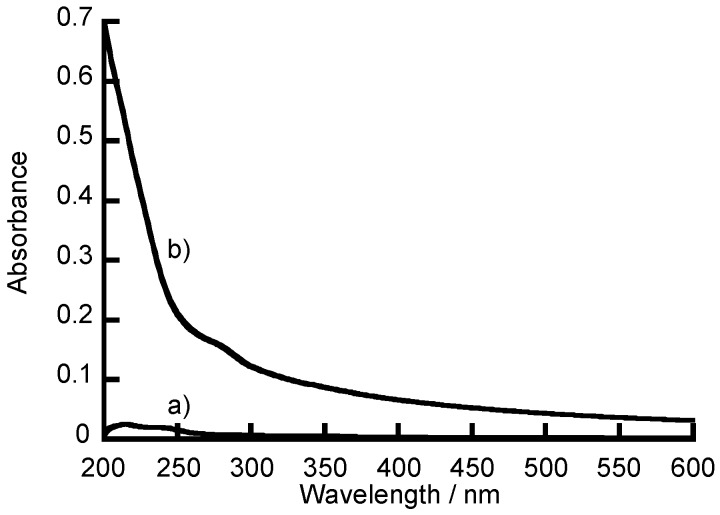
UV-visible absorption spectra for the (NAF/PAH)_5_NAF film (**a**) before and (**b**) after exposure to a 1 mg mL^−1^ insulin solution (pH 7.4) for 12 h.

Figure 4a,b show UV-vis absorption spectra for the (NAF/PAH)_5_NAF film before and after exposure to a 1 mg mL^−1^ insulin solution in 10 mM Tris buffer containing 150 mM NaCl (pH 7.4) for 12 h, respectively. The LbL film immersed in the insulin solution exhibited a slight absorption at 280 nm due to insulin. However, insulin adsorbed on the LbL film was widely turbid and not suitable for quantitative analysis of the insulin loading in the LbL film. Previous studies have also reported that insulin in the LbL films could not be quantified due to turbidity [11,26]. The disappearance of the turbidity in the adsorbed-insulin was confirmed by the absorption spectrum after the insulin-adsorbed LbL films were exposed to a 0.1 M sodium hydroxide solution for 5 h. The NAF undergoes ion exchange with sodium ions [35], and the positive charges of PAH are weakened by the strongly alkaline solution. Therefore, the LbL films decompose and lose affinity by electrostatic attraction between the layers when the film-coated glass slides were exposed to sodium hydroxide solution [36]. The loading of insulin on the LbL films was thus determined from the absorbance of LbL films dissolved in sodium hydroxide solution.

**Figure 5:**
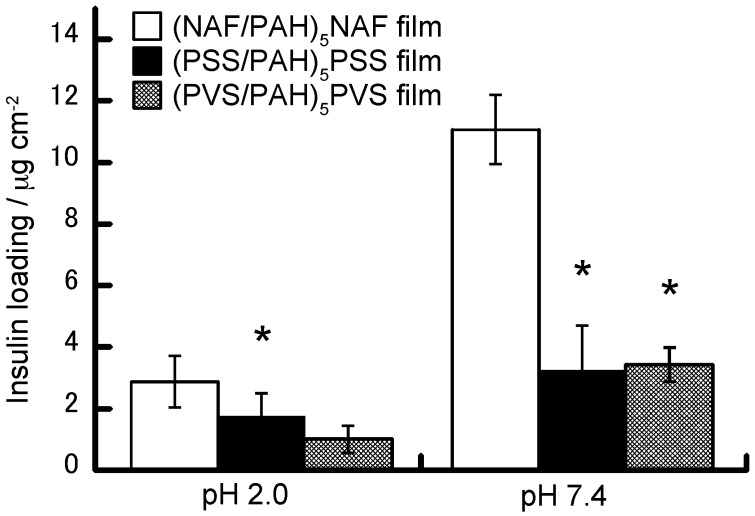
Insulin loading on (NAF/PAH)_5_NAF (open columns), (PSS/PAH)_5_PSS (filled columns), and (PVS/PAH)_5_PVS (half-filled columns) films. The LbL films were exposed to a 0.1 mg mL^−1^ insulin solution with 10 mM acetate buffer (pH 2) or 10 mM Tris buffer (pH 7.4). All buffer solutions contained 150 mM NaCl. * *p* < 0.05 one-way analysis of variance (ANOVA) and Dunnett’s test vs. (NAF/PAH)_5_NAF film. Note: Poly(styrene sulfate) (PSS) and poly(vinylsulfonate) (PVS).

Figure 5 shows the amounts of insulin loaded onto the (NAF/PAH)_5_NAF, (PSS/PAH)_5_PSS, and (PVS/PAH)_5_PVS films. Previous reports have shown that it is possible to prepare PSS-PAH and PVS-PAH LbL films [1,37]. The purpose of this study was to investigate insulin-adsorbed LbL films using NAF, which has a higher specific adsorption for insulin than each polyanion. The amount of insulin loading was calculated to be 2.88 ± 0.84 µg cm^−2^ for the (NAF/PAH)_5_NAF film, 1.75 ± 0.75 µg cm^−2^ for the (PSS/PAH)_5_PSS film, and 1.01 ± 0.44 µg cm^−2^ for the (PVS/PAH)_5_PVS film, when the LbL films were exposed to a 0.1 mg mL^−1^ insulin solution at pH 2.0. It is probable that positively charged insulin is adsorbed onto the polyanion layers in the LbL films by electrostatic affinity because the isoelectric point (*pI*) of insulin was reported to be 5.4 [38] and the polyanions are negatively charged at pH 2.0. The amount of insulin loading was also dependent on the type of polyanion. On the other hand, the loading of insulin in (NAF/PAH)_5_NAF films exposed to insulin solution at pH 7.4 was calculated to be 11.07 ± 1.12 µg cm^−2^, which is higher than that of the other LbL films. Insulin (*pI* 5.4) at pH 7.4 has both a negative charge and a partial positive charge within molecules, which suggests the adsorption of insulin to PAH and partial adsorption to the polyanion layers.

**Figure 6:**
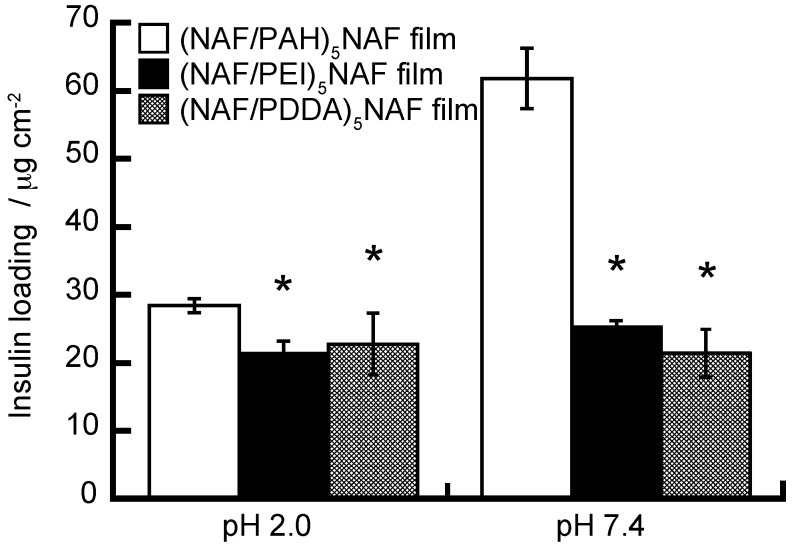
Insulin loading on (NAF/PAH)_5_NAF (open columns), (NAF/PEI)_5_NAF (filled columns), and (NAF/PDDA)_5_NAF (half-filled columns) films. The LbL films were exposed to a 1 mg mL^−1^ insulin solution with 10 mM acetate buffer (pH 2) or 10 mM Tris buffer (pH 7.4). All buffer solutions contained 150 mM NaCl. * *p* < 0.05 one-way ANOVA and Dunnett’s test vs. (NAF/PAH)_5_NAF film.

We have evaluated the amounts of insulin loaded onto (NAF/PAH)_5_NAF, (NAF/PEI)_5_NAF, and (NAF/PDDA)_5_NAF films, as shown in Figure 6, considering whether different polycations result in the adsorbtion of different amounts of insulin. The loading of insulin was higher in the NAF-PAH LbL film than in the NAF-PEI and NAF-PDDA LbL films exposed to the insulin solution. This is probably due to the dependence of the amount of insulin loading on the deposition amount and thickness of the LbL films. The concentration of the insulin solution used in relation to the results in Figure 6 was higher than that used in relation to the results in Figure 5. The amount of insulin loaded onto the (NAF/PAH)_5_NAF film increased at higher insulin concentrations. The amount of insulin loading on the LbL films was higher at pH 7.4 than at pH 2.0, which suggests that the net charge of insulin is more likely to promote adsorption onto a positively charged membrane than a negatively charged membrane.

Table 1 summarizes the insulin loading in the LbL films as a function of the number of layers. Insulin loading was evaluated for polycation- and NAF-terminated films with five and ten bilayers. The insulin loading of the film increased with the number of bilayers. In this context, the loading of closely packed insulin molecules in a monomolecular layer on a flat surface has been reported to be 0.11 μg cm^−2^ [39]. Despite the surface roughness of the LbL films, the amount of insulin adsorption is too great to be adsorbed onto only the surface of the LbL films. Therefore, insulin is not only adsorbed onto the surface of the LbL films but is also absorbed into the interior of the LbL film. The insulin loading in the NAF-PAH films was higher than that of the other films. The higher loading of insulin in each layer is likely due to the formation of an aggregated form of insulin in the LbL film. Insulin is known to form multimeric complexes in solution and on solid surfaces with the size of the aggregates found to be dependent on the composition of the solution [39,40]. 

**Figure 7:**
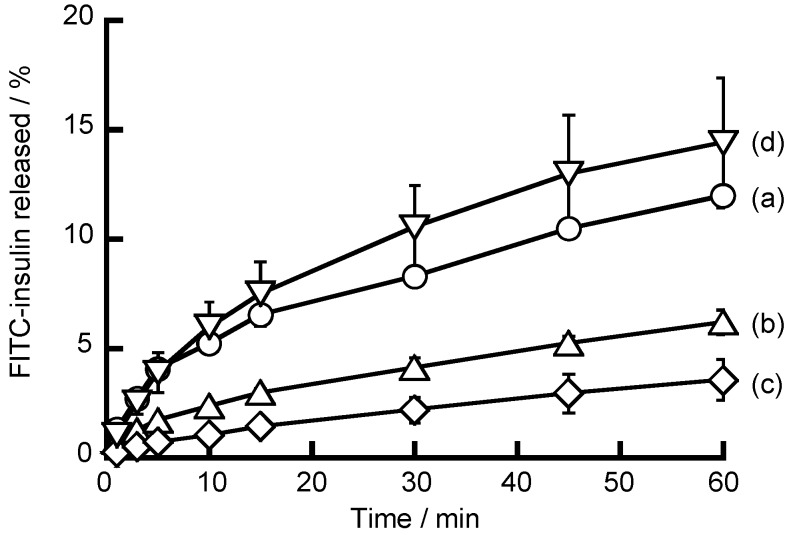
Fluorescein isothiocyanate-labeled insulin (FITC-insulin) release from (NAF/PAH)_5_NAF film in buffer solutions at (**a**) pH 2.0; (**b**) pH 4.0; (**c**) pH 7.4; and (**d**) pH 9.0.

The release of FITC-insulin from (NAF/PAH)_5_NAF films was studied using fluorescence spectroscopy, as shown in Figure 7. It was expected that the net electric charge of insulin would change with the pH and that insulin adsorbed in the LbL films would be released. The net electric charge of insulin (*pI* of insulin is 5.4) is positive at pH 7.4 and pH 9.0, and negative at pH 2.0 and pH 4.0. (NAF/PAH)_5_NAF films exposed to an insulin solution at pH 7.4 are expected to adsorb insulin onto PAH and partially adsorb insulin onto the polycation in the LbL film. When the LbL films are immersed in an acidic solution (pH < 5.4) of insulin, it is expected that FITC-insulin would be released from the LbL films due to a loss of the electrostatic affinity. The release of FITC-insulin from (NAF/PAH)_5_NAF films was low at pH 4.0 and pH 7.4. It is suggested that insulin adsorbed on (NAF/PAH)_5_NAF films forms sparingly soluble aggregates at pH 4.0 and pH 7.4, thereby causinginsulin release to be slow. In contrast, more FITC-insulin was released from (NAF/PAH)_5_NAF films at pH 2.0 and pH 9.0. Insulin release was promoted at pH 2.0 because the negative charge of insulin disappears due to the protonation of insulin, resulting in its release from each of the PAH layers owing to the loss of the electrostatic affinity. For alkaline insulin solutions, at pH 9.0 the attraction between the positive charge of the amino groups in insulin and the negative charge of the PAH layers weakens, resulting in a tendency for insulin to be released from the LbL films. Insulin release from LbL films with NAF requires consideration of the influence of the functional groups possessed by insulin and the polymer, rather than the net charge of insulin.

Table 2 summarizes the percentage of FITC-insulin release from (NAF/PAH)_5_NAF, (NAF/PEI)_5_NAF, and (NAF/PDDA)_5_NAF films. The percentage released was calculated from the solution at each pH of the LbL films after immersion for 60 min. The percentage of insulin released from the NAF-PEI and NAF-PDDA films was higher than that of the NAF-PAH film. It is conceivable that the percentage of FITC-insulin released is dependent on the thickness of the LbL film. When the NAF-PEI and NAF-PDDA films were immersed in solutions at pH 2.0 and pH 9.0, there was an increase in the percentage of FITC-insulin released compared with that from the NAF-PAH film. PEI has branched chains and is composed of fewer primary amine groups (-NH_2_) than PAH. Therefore, the number of positive charges on PEI is less than that on PAH, resulting in a lower adsorption of insulin due to electrostatic affinity in the PEI layer compared to the PAH layer. While insulin adsorbed on the NAF-PAH film forms a sparingly soluble aggregate, it is presumed that this is easily released due to the weak adsorption of insulin onto the NAF-PEI films. PDDA is an extended chain compound compared with PAH; therefore, PDDA adsorbs flatly onto the LbL film compared with PAH. Consequently, it is presumed that steric hindrances in the NAF-PDDA film are small and that insulin can be easily released.

## 4. Conclusions

LbL films comprised of NAF and a polycation were successfully prepared by exploiting the electrostatic affinity of the components. When an LbL film was immersed in an insulin solution, insulin was adsorbed onto the LbL film. The amount of insulin adsorbed was highest for the NAF-PAH film at pH 7.4. The amount of insulin loading increased with the number of bilayers in the LbL film. The release of insulin from LbL films was accelerated at pH 2 and pH 9 but was suppressed at pH 4 and pH 7.4 due to the electrostatic affinity between insulin and the LbL films. Insulin was adsorbed in the LbL films with NAF under mild conditions; therefore, these LbL films could be used as drug reservoirs for DDSs.

## Figures and Tables

**Table 1:** Amount of insulin loading in LbL films.

Loading of Insulin in LbL Films/µg cm^−2^				
	pH 7.4		pH 2.0	
LbL film	n = 5	n =10	n = 5	n = 10
(NAF/PAH)_n_	61.8 ± 7.4	105.6 ± 2.8	27.1 ± 3.0	40.4 ± 2.7
(NAF/PAH)_n_NAF	61.8 ± 4.5	97.8 ± 4.1	28.5 ± 1.0	39.4 ± 3.0
(NAF/PEI)_n_	24.3 ± 3.3	45.7 ± 3.7	20.2 ± 0.3	24.2 ± 3.7
(NAF/PEI)_n_NAF	25.3 ± 0.9	43.6 ± 0.7	21.4 ± 1.8	25.1 ± 0.7
(NAF/PDDA)_n_	20.8 ± 2.0	23.6 ± 0.6	16.8 ± 2.0	19.2 ± 1.3
(NAF/PDDA)_n_NAF	21.4 ± 3.5	24.5 ± 1.1	22.8 ± 4.5	17.0 ± 1.1

The LbL films were exposed 1 mg mL^−1^ insulin with 10 mM acetate buffer (pH 2.0) or 10 mM Tris buffer (pH 7.4). All buffer solutions contained 150 mM NaCl.

**Table 2:** Amount of FITC-insulin released from LbL films.

FITC-Insulin Released/%				
LbL film	pH 2.0	pH 4.0	pH 7.4	pH 9.0
(NAF/PAH)_5_NAF	12.0 ± 0.4	6.2 ± 0.6	3.6 ± 0.9	14.4 ± 3.0
(NAF/PEI) _5_NAF	54.4 ± 14.3	23.8 ± 12.4	7.4 ± 0.9	62.0 ± 10.6
(NAF/PDDA)_5_NAF	44.0 ± 1.7	25.1 ± 2.9	21.6 ± 1.7	69.8 ± 0.1

The LbL films were exposed 0.1 mg mL^−1^ FITC-insulin solution (pH 7.4). The percentage of FITC-insulin released was calculated from the solution at each pH immersion of the films for 60 min.
